# Localization and expression of ADAM2 in the dromedary camel testis, epididymis and sperm during rutting season

**DOI:** 10.1590/1984-3143-AR2020-0241

**Published:** 2021-04-07

**Authors:** Abdulkarem Al-Shabebi, Thnaian Althnaian, Khalid Alkhodair

**Affiliations:** 1 Department of Anatomy, College of Veterinary Medicine, king Faisal University, Al-Ahsa, Saudi Arabia; 2 College of Veterinary Medicine, Thamar University, Dhamar, Yemen

**Keywords:** camel, fertility, immunohistochemistry, gene expression

## Abstract

ADAM2 (fertilin β) is a sperm surface protein reported in several mammalian species. However, the presence of ADAM2 in the male reproductive system and sperm of the camel is not well known. The present study was to clarify the localization and expression of ADAM2 in the dromedary camel testis, epididymis and spermatozoa during rutting season using immunohistochemistry (IHC) and the quantitative real-time polymerase chain reaction (qPCR). Tissue samples were obtained from the testis (proximal and distal) and epididymis (caput, corpus, and cauda) from eight mature male camels. Epididymal and ejaculated sperms were collected from four other fertile camels. IHC analysis clearly showed the localization of ADAM2 protein in the spermatocytes and the round and elongated spermatids of the testis, in the epithelial cells along the epididymis tract, on the posterior head of the sperm within the cauda epididymis, and on the acrosomal cap of both the epididymal and ejaculated sperm. The expression of camel ADAM2 mRNA was significantly higher (*P* < 0.05) in the testis when compared with the epididymis. These findings may suggest an important role of ADAM2 in the fertility of male dromedary camels.

## Introduction

Modern breeding programs in domestic animal farms use artificial insemination to improve the genetic and economic attributes of the animals. Although the classic semen parameters such as motility and viability are normal, there is still low fertility in some males (Fàbrega et al., 2011). For that reason, molecular tools have been developed to estimate the fertility levels precisely by estimating the levels of expression of certain sperm proteins that are used as markers of male fertility (Ashrafzadeh et al., 2013). ADAM2 is an integral membrane protein of the ADAM (A Disintegrin And Metalloproteinase) family that plays vital roles in male reproduction. The role was studied earlier than those of the other members of the reproductive ADAM proteins (Choi et al., 2016). Earlier studies in guinea pigs showed that ADAM2 was synthesized in the testis and processed in the epididymis (Blobel et al., 1990). The transcription of ADAM2 was detected in the testes of several mammalian species including bulls (Waters and White, 1997), pigs (Fàbrega et al., 2011), monkeys (Perry et al., 1995), rabbits (Hardy and Holland, 1996), rats (McLaughlin et al., 1997), mice (Han et al., 2009) and also in humans (Choi et al., 2016). Immunohistochemistry (IHC) analysis revealed that the distribution of ADAM2 was along the head of the testicular sperm, while it was concentrated on the posterior part of the epididymal sperm (Hunnicutt et al., 1997). In addition, this protein was constantly found on acrosome-intact sperm but becomes very mobile in acrosome-reacted sperm at the plasma membrane (Oh et al., 2005). Chen et al. (1999) predicted the contribution of ADAM2 to the mediation of the sperm-egg membrane interactions by its bounding of the integrin α6β1 receptor of oolemma with an assisting CD9 protein. An evident role of ADAM2 in the fertility of males was suggested by a study that involved the deletion of ADAM2 gene in the mouse sperm, which led to male infertility due to impaired sperm migration inside the female reproductive tract and decreased the ability of sperm to merge with the oocyte (Cho et al., 1998).

Due to lack of studies on ADAM2 protein in camels and the importance of this protein in male fertility, this work was undertaken to study the presence and expression of ADAM2 in the dromedary camel testis and epididymis, as well as its localization on spermatozoa during rutting season to help fill in the gap in knowledge about this protein in camels.

## Materials and methods

### Experimental materials (animals and tissues)

Testis and epididymis were collected from 12 clinically healthy adult dromedary camels (local breed, 6-15 years of age) from a local abattoir in Al-Ahsa, in the eastern part of Saudi Arabia during local rutting season (November to April). After slaughter, macroscopic inspection was done to ensure sampling from clinically normal reproductive organs. Immediately following evisceration, samples from collected testis and epididymis (n = 8) were placed in 10% buffered formalin for 36 hours for immunohistochemistry (IHC). Other samples from the same organs (n = 8) were fresh-frozen in liquid nitrogen within 10 minutes of evisceration and kept at –80 °C. These samples were processed at 4 °C for the quantitative polymerase chain reaction analysis (qPCR). Tissue specimens were collected from the testes (proximal and distal) and epididymis (caput, corpus, and cauda) of eight genital systems. Semen was collected from the cauda epididymis of the other four males as described previously by Alkhodair et al. (2018). Under the approved guidelines for the ethical treatment of animals and as described earlier by Tingari et al. (1986), ejaculated semen samples were collected from four mature, fertile dromedary healthy camels of local breed (obtained from the Camel Research Center, at King Faisal University, Al-Ahsa, Saudi Arabia). Samples were washed by phosphate buffered saline (PBS) and centrifuged twice at 2000 rpm for 5 min at room temperature (RT). After that, sperms were counted using a Makler chamber and diluted to approximately 20 x 10^4^/ml in PBS. Sperms were then smeared on Superfrost® slides, air-dried, and stored at –20 °C until use.

### Immunohistochemistry

#### Tissue immunostaining

For tissue immunostaining, formalin-fixed samples were dehydrated through an ethanol series, cleared in xylene, and embedded in paraffin wax. Sections of 4 µm thick were cut with a microtome (Lica, Germany) and placed on Superfrost slides (VWR, cat. no. 631-0108). After dewaxing and rehydration, sections were stained with rabbit polyclonal anti-human ADAM2 antibody (1:100) (Sigma-Aldrich, USA, cat. no. HPA026581) using the avidin–biotin–peroxidase complex method (Hsu et al., 1981; Adeghate et al., 2001). Due to the lack of the camel ADAM2 antibody, the polyclonal antibody against human ADAM2 (Prestige Antibody®, Sigma-Aldrich, Cat. No. HPA026581) was alternatively employed. Moreover, we conducted sequence comparisons on multiple-sequence alignments of ADAM2 of *Camelus dromedarius* and *Homo sapiens* protein-protein BLAST (Blastp) at NCBI (2020a). These analyses showed significant conservation across species along the epitope areas.

Antigen-retrieval buffer (sodium citrate 50 mM, pH 6.0) was prepared to enhance the epitope access immunostaining signal at 100 °C for 20 min. Tissue slides were incubated with 90 μL of 3% hydrogen peroxide for 10 min, normal goat serum (NGS) (Abcam, Inc., cat. no. ab7481) (1:20 dilution) for 10 min, and diluted primary antibody (dilution 1:100) for 1 hour at RT. The suitable biotinylated secondary antibody (1:100) was then added, and slides were then incubated for 30 min at RT. Streptavidin HRP (Abcam, Inc., cat. no. ab64269) was added to each slide for 20 min. Color was developed by adding a suitable amount of 3,3’-diaminobenzidine tetrahydrochloride chromogen substrate for 5 min. Washing steps between each reagent were performed using PBS. Slides were counterstained with hematoxylin, dehydrated and cleared, placed on a cover slip using a synthetic mounting (DPX), and visualized using a light microscope (Microscope Leica DM6000 B, Germany).

#### Sperm immunostaining

Fluorescent immunostaining was conducted to localize and visualize the expression of ADAM2 on ejaculated and epididymal sperms using fluorescent microscopy. Sperm-smeared slides were surrounded by the PAP pen (Abcam, Inc., cat. no. ab2601). Slides were incubated in PBS for 5 min, 70 μL of normal goat serum (NGS) (Abcam, Inc., cat. no. ab7481) (1:20 dilution) was added, and incubation took place for 10 min at RT. Then, 100 μL of primary antibody (dilution 1:100) was added to each slide and incubated for 1 hour at RT. PBS washing was performed three times for 5 min for each one of the slides. After that, goat anti-rabbit fluorescent secondary antibody (FITC) (Abcam, Inc., cat. no. ab6717) (dilution 1:100) was added and the slides were then incubated for 2 hours in a dark place at RT. Washing steps between each reagent were performed on the slides, and a cover slip was placed over the slides using aqueous non-fluorescent mounting medium. The slides were examined and visualized immediately using a fluorescence microscope (Fully Automated Upright Microscope Leica DM6000 B, Germany). While chromogenic immunostaining of sperm was performed similar to that conducted on tissue slides.

### RNA extraction and quantitative real-time polymerase chain reaction (qPCR)

Gene-specific primers were designed to amplify the dromedary camel ADAM2 and glyceraldehyde 3-phosphate dehydrogenase (GAPDH) genes using the NCBI (2020b) primer-blast website. Primer sequences are listed in Table 1.

**Table 1:** Primer sequences designed and employed to amplify ADAM2 and glyceraldehyde 3-phosphate dehydrogenase (GAPDH) genes of the dromedary camel.

**Gene name**	**Primer sequences**	**Gene ID, accession, number**	**Amplicon Length (bp)**
ADAM2	F: TGA GTG GGG CAA TCC AAT GT	XM_010999136.1	140
	R: TTC GCA CTT CGT GTA CCC TG		
GAPDH	F: CCT GGA GAA ACC TGC CAA ATA	XM_010990867.2	207
	R: TCG TTG TCG TAC CAG GAA ATG		

A tissue sample of 50 mg was collected and homogenized using a Bead Ruptor Homogenizer (OMNI International, NW Kennesaw, GA, USA). TRIzol® Reagent (Invitrogen, Carlsbad, CA, USA) was used to extract the total RNA as described by the manufacturer. The total RNA was precipitated using chloroform and isopropanol and resuspended in ultrapure diethylpyrocarbonate-(DEPC)-treated RNase-free water (Invitrogen, USA). The total RNA was then analyzed for concentration and purity using the BioTek Synergy HTX reader (BioTek, USA).

RNA was reverse-transcribed to cDNA using the iScript cDNA Synthesis Kit (BioRad, Hercules, CA, USA) in 20 μL volume of total mixture with 4 μL iScript® Reaction Mix, 1 μL iScript® Reverse Transcriptase, and nuclease-free distilled water. The mixed solution was inculpated at 25 °C for 5 min, 46 °C for 20 min, and thereafter at 95 °C for 60 seconds to inactivate the reverse-transcriptase.

qPCR reaction for each sample was carried out by the CFX96® Touch Real-time PCR (BioRad, USA) using the SsoAdvanced SYBR Green Supermix (BioRad, USA). Briefly, the total reaction mixture volume of 20 μL contained 10 μL master mix, 2 μL forward primer (10pmol) 2 μL reverse primer (10pmol), and 2 μL cDNA template, and 4 μL nuclease-free water. The thermal cycling conditions were: 95 °C for 30 s; 40 cycles of 95 °C for 15 s, and after that 60 °C for 30 s and 72 °C for 10 s. All cDNA templates were run in duplicate, and fluorescence emission was detected. The relative quantifications of expressed ADAM2 gene were automatically calculated via CFX Manager™ software V3.1 (BioRad, Hercules, CA, USA) and by using the housekeeping GAPDH gene as a reference.

### Statistical analysis

Data were analyzed using SPSS® software version 16. Comparisons were made among different tissues with varying expressions as means ± the standard errors via a one-way analysis of variance with post hoc analysis. Data were shown as means ± standard errors.

## Results

### Immunolocalization of ADAM2 in the testis and epididymis

**Figure 1:**
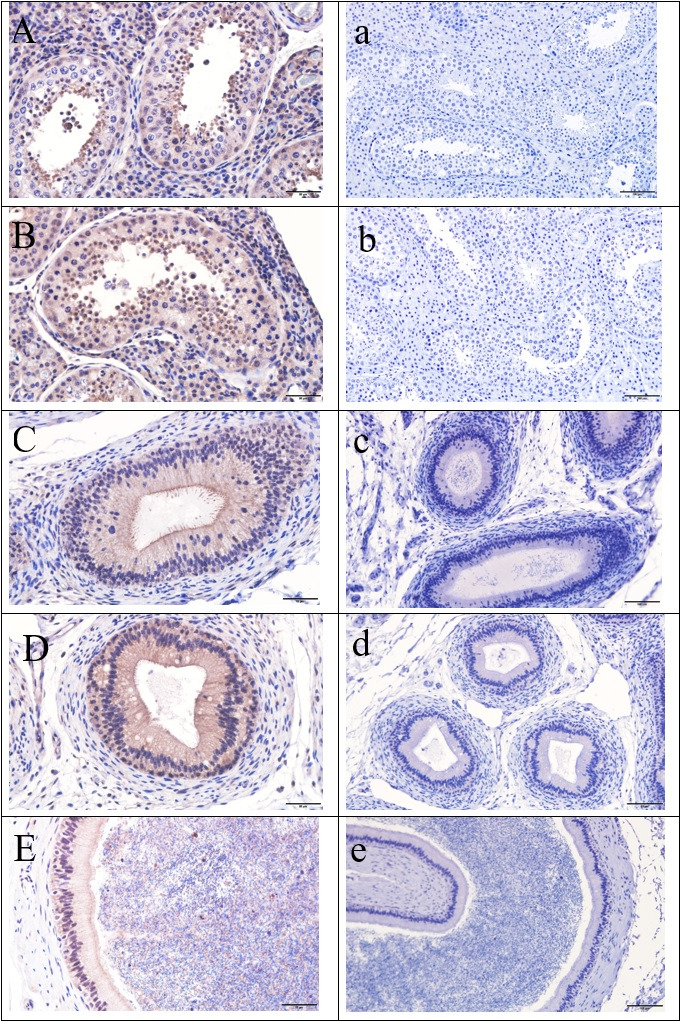
Immunohistochemical localization of the ADAM2 protein in the testis and epididymis of dromedary camels. (A) Proximal testis; (B) distal testis; (C) caput epididymis; (D) corpus epididymis; and (E) cauda epididymis (40x, scale bar, 50 μm). The brown color indicates the presence of ADAM2 protein in the sections with blue contrasting (hematoxylin); (a-e) Sections of the same tissues were incubated with normal rabbit serum instead of primary antibody as control samples (20x, scale bar, 100 μm).

ADAM2 immunolabeling was observed in all of the examined parts of the testis and epididymis with different intensities. In the testis, a highly intense staining was detected in spermatocytes and round and elongating spermatids, a lesser staining was observed in the spermatogonia of the seminiferous tubules at the proximal and distal parts, and a moderate staining was shown in the Leydig cells within interstitial tissue (Figure 1A and B). In the caput, corpus, and cauda of the epididymis, a moderate staining was observed in the cytoplasm of the epithelial cells (Figure 1C, D, and E), whereas in the caudal region of epididymis, the reaction was shown on the acrosomal part of the sperm (Figure 1E).

### Fluorescent and chromogenic immunostaining of camel sperm

**Figure 2:**
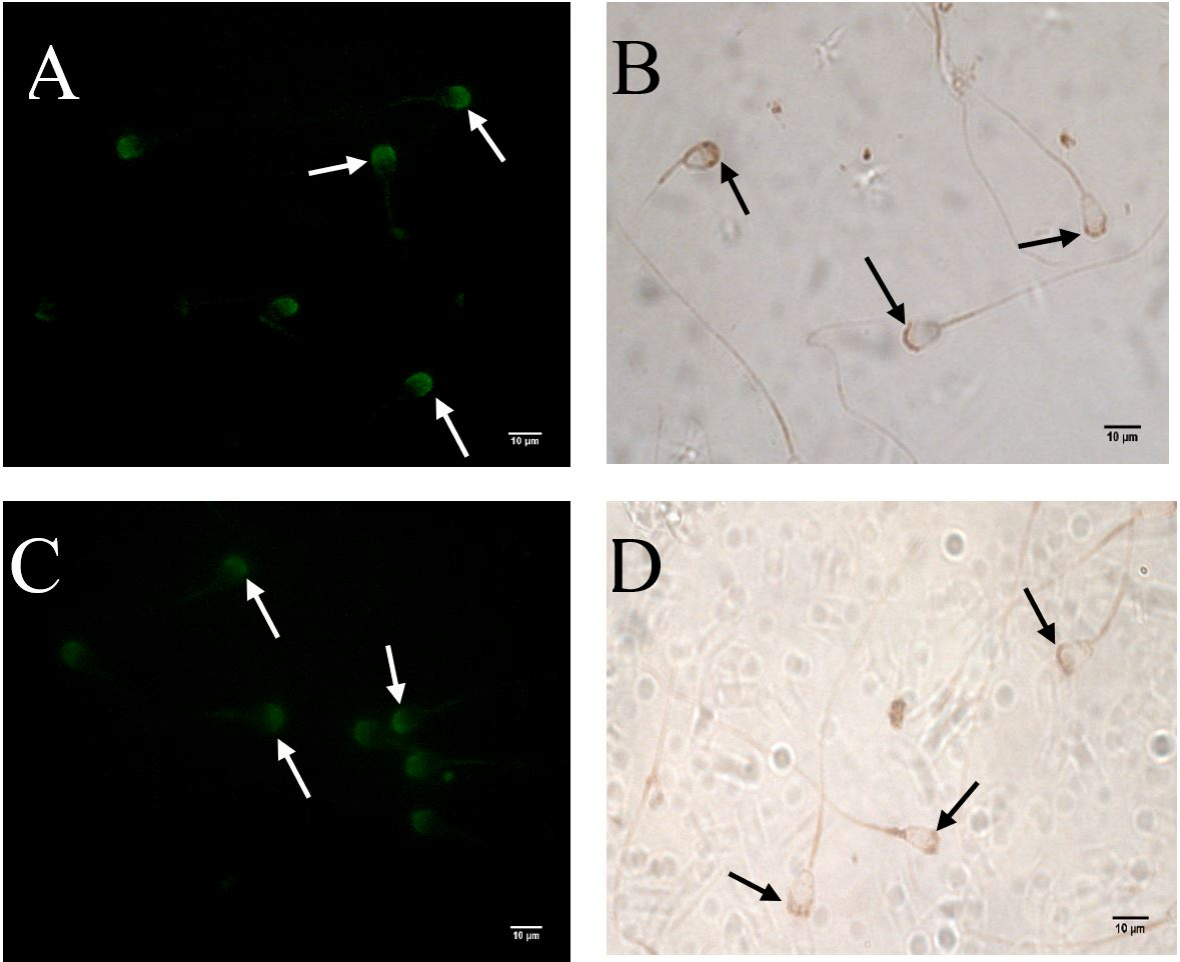
Fluorescent and chromogenic immunostaining of ADAM2 protein on the mature camel sperm. Arrows show the immunostaining of ADAM2 protein on sperms. (A, B) Ejaculated sperm; (C, D) epididymal sperm (60x, scale bar=10 μm).

ADAM2 protein was also found in ejaculated and epididymal sperms by fluorescent and chromogenic immunostaining methods. However, the ejaculated sperm exhibited a higher level of staining (Figure 2A and B) than in the epididymal sperm (Figure 2C and D). Specific staining was evident on the acrosomal cap in most of the examined sperms. No obvious staining was detected in other parts of the sperm.

### Relative gene expression of ADAM2 in the testis and epididymis

**Figure 3:**
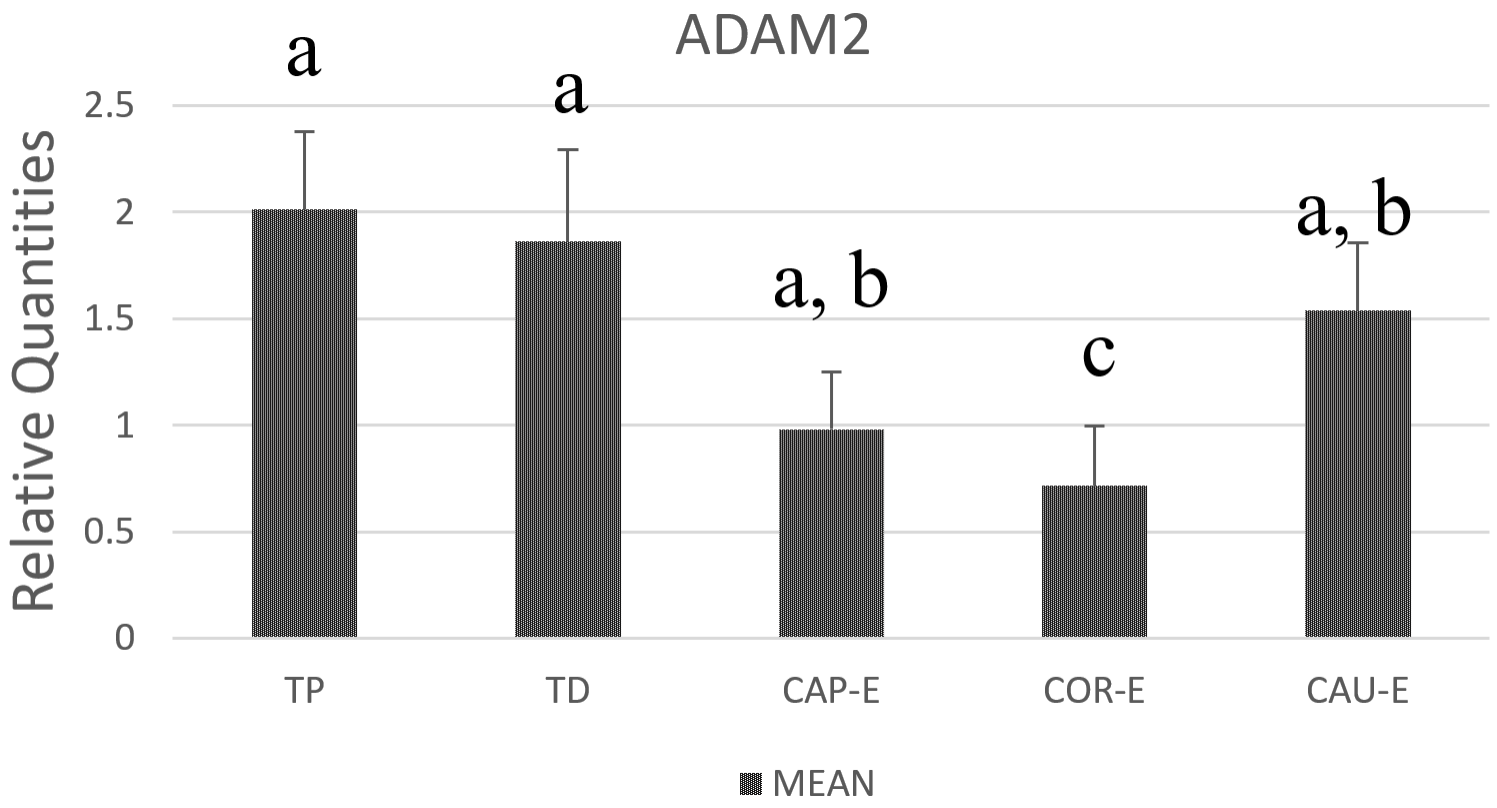
Relative quantification of ADAM2 mRNA in the testis and epididymis of dromedary camels: TP, proximal testis; TD, distal testis; CAP-E, caput epididymis; COR-E, corpus epididymis; CAU-E, cauda epididymis. The gene expressions were quantified in relation to GAPDH and showed as means ± standard errors. The significance was set to P < 0.05, and the different letters (a, b, c) indicate the significance among examined tissues. Parts of the testes showed a high expression in comparison with other parts of the epididymis.

To complement the protein expression, RNA transcription levels of ADAM2 were examined in the tissues of the testis and epididymis. All examined parts showed an expression for ADAM2 mRNA at various levels (Figure 3). The data indicated that there was a significant higher expression levels of ADAM2 in all parts of the testis than in the epididymis (*P* <0.05). The expression of ADAM2 was higher in the cauda of the epididymis, while an intermediate expression was detected in the caput of the epididymis. The lowest expression was in the corpus of the epididymis.

## Discussion

Several studies reported the presence of ADAM2 protein in the testis and epididymis of some mammalian species (Perry et al., 1995; Hardy and Holland, 1996; Lum and Blobel 1997; McLaughlin et al., 1997; Waters and White, 1997; Han et al., 2009; Fàbrega et al., 2011; Choi et al., 2016). To the best of the authors knowledge, there was no published studies showing the presence of ADAM2 protein and/or ADAM2 mRNA in the testis, epididymis, and sperms of dromedary camels. In this study we managed to show the presence of ADAM2 protein within the testis, epididymis, and sperms using IHC and mapped its expression using qPCR in dromedary camels for the first time. The ability of the human ADAM2 antibody to recognize ADAM2 protein in camels was confirmed by pilot trials of immunostaining camel sperms, which gave specific immunoreactivity on the employed sperms (data not shown).The currently revealed localization of ADAM2 protein in spermatocytes and in round and elongating spermatids within seminiferous tubules of dromedary camel similar findings in the human testis (www.proteinatlas.org), both of which were studied using the Antibody (Sigma-Aldrich Cat. no. HPA026581). The elongated spermatids of the rat testes also showed localization of ADAM2 protein (Lum and Blobel, 1997). In this study, lower level of ADAM2 protein staining was observed in Leydig cells. In another study in humans, a positive correlation was observed between ADAM2 expression and the level of testosterone (Zalata et al., 2016). The previously reported hypertrophy with increase in the number of Leydig cells of camels during rutting season (Gherissi et al., 2018) may explain the currently reported increased levels of ADAM2 protein in Leydig cells in this study. In mice, rats, and monkeys, ADAM2 protein are initially synthesized in testicular germ cells, which may indicate an early role of this protein in the function and/or development of the sperm (Kim et al., 2010; Choi et al., 2016). The latter findings are supported by the findings of the current study, which managed to localize the presence of ADAM2 protein in cells involved in the early stages of sperm development such as the spermatogonia, spermatocytes, round and elongated spermatids of seminiferous tubules, and in Leydig cells. Previous studies also reported the expression of ADAM2 protein in the epididymis of monkeys (Cho et al., 2000), boars (Fàbrega et al., 2011) and mice (Kim et al., 2010). These findings are in agreement with the findings of the present study, which showed intense staining of ADAM2 protein in the epithelia layer of the epididymis of the camel.

The presence of ADAM2 protein in the sperm was quantitatively reported in cattle (Waters and White, 1997), guinea pigs (Fàbrega et al., 2011), monkeys (Kim et al., 2010), mice (Marcello and Evans, 2010) and rats (Lum and Blobel, 1997). In this study, the presence of ADAM2 protein exhibited highly intense staining in the acrosomal cap of the camel sperm, similar to that reported from of the rat (Lum and Blobel, 1997). In contrast, Yuan et al. (1997) reported the restricted presence of ADAM2 protein at the equatorial region of mouse sperm, while Primakoff et al. (1987) reported the localization of ADAM2 protein at the posterior head region of guinea pig sperm. Interestingly, ADAM2 protein was not detected in human sperm (Choi et al., 2016). The currently reported higher levels ADAM2 immunostaining in ejaculated sperm comparison to that in epididymal sperm may suggest further expression of the protein to the sperms while travelling in the camel reproductive tract away the epididymis such as the male accessory glands.

The level of the ADAM2 mRNA in the male genital organs of mammals has previously received little attention. High levels of ADAM2 mRNA were once reported in the testes of boars (Fàbrega et al., 2011) and rats (Lum and Blobel, 1997) and in lower levels in the epididymis of boar (Fàbrega et al., 2011). These findings are supported by the currently reported high expression of ADAM2 mRNA in the testis and lesser expression in the epididymis. The latter finding is in agreement with the currently found IHC profile, indicating a variation in the expression of this protein in the sperms depending on the localization and maturation of the sperms within the camel reproductive tract.

## Conclusion

The result of the current study evidently indicates the presence of ADAM2 protein in the camel testis, epididymis, and sperm. The current results suggest a possible role of ADAM2 protein in the development of the sperm within the male camel reproductive system from early sperm formation in the testis to later sperm formation and maturation prior ejaculation. Furthermore, the localization of ADAM2 protein on the acrosomal cap may suggest a probable role in the later fertilization process in camels.

## References

[B001] Adeghate E, Ponery AS, Pallot DJ, Singh J (2001). Distribution of vasoactive intestinal polypeptide, neuropeptide-Y and substance P and their effects on insulin secretion from the in vitro pancreas of normal and diabetic rats. Peptides.

[B002] Alkhodair K, Almhanna H, McGetrick J, Gedair S, Gallagher ME, Fernandez-Fuertes B, Tharmalingam T, Larsen PB, Fitzpatrick E, Lonergan P, Evans ACO, Carrington SD, Reid CJ (2018). Siglec expression on the surface of human, bull and ram sperm. Reproduction.

[B003] Ashrafzadeh A, Karsani SA, Nathan S (2013). Mammalian sperm fertility related proteins. Int J Med Sci.

[B004] Blobel CP, Myles DG, Primakoff P, White JM (1990). Proteolytic processing of a protein involved in sperm-egg fusion correlates with acquisition of fertilization competence. J Cell Biol.

[B005] Chen MS, Tung KSK, Coonrod SA, Takahashi Y, Bigler D, Chang A, Yamashita Y, Kincade PW, Herr JC, White JM (1999). Role of the integrin-associated protein CD9 in binding between sperm ADAM 2 and the egg integrin α6β1: implications for murine fertilization. Proc Natl Acad Sci USA.

[B006] Cho C, Bunch DO, Faure J-E, Goulding EH, Eddy EM, Primakoff P, Myles DG (1998). Fertilization defects in sperm from mice lacking fertilin β. Science.

[B007] Cho C, Ge H, Branciforte D, Primakoff P, Myles DG (2000). Analysis of mouse fertilin in wild-type and fertilin β−/− sperm: evidence for C-terminal modification, α/β dimerization, and lack of essential role of fertilin α in sperm-egg fusion. Dev Biol.

[B008] Choi H, Jin S, Kwon JT, Kim J, Jeong J, Kim J, Jeon S, Park ZY, Jung KJ, Park K, Cho C (2016). Characterization of mammalian ADAM2 and its absence from human sperm. PLoS One.

[B009] Fàbrega A, Guyonnet B, Dacheux J-L, Gatti J-L, Puigmulé M, Bonet S, Pinart E (2011). Expression, immunolocalization and processing of fertilins ADAM-1 and ADAM-2 in the boar (Sus domesticus) spermatozoa during epididymal maturation. Reprod Biol Endocrinol.

[B010] Gherissi DE, Afri-Bouzebda F, Bouzebda Z (2018). Seasonal changes in the testicular morphology and interstitial tissue histomorphometry of Sahraoui camel under Algerian extreme arid conditions. Biol Rhythm Res.

[B011] Han C, Choi E, Park I, Lee B, Jin S, Kim DH, Nishimura H, Cho C (2009). Comprehensive analysis of reproductive ADAMs: relationship of ADAM4 and ADAM6 with an ADAM complex required for fertilization in mice. Biol Reprod.

[B012] Hardy CM, Holland MK (1996). Cloning and expression of recombinant rabbit fertilin. Mol Reprod Dev.

[B013] Hsu S, Raine L, Fanger H (1981). The use of antiavidin antibody and avidin-biotin-peroxidase complex in immunoperoxidase technics. Am J Clin Pathol.

[B014] Hunnicutt GR, Koppel DE, Myles DG (1997). Analysis of the process of localization of fertilin to the sperm posterior head plasma membrane domain during sperm maturation in the epididymis. Dev Biol.

[B015] Kim E, Lee J-W, Baek DC, Lee S-R, Kim M-S, Kim S-H, Kim CS, Ryoo ZY, Kang HS, Chang KT (2010). Processing and subcellular localization of ADAM2 in the Macaca fascicularis testis and sperm. Anim Reprod Sci.

[B016] Lum L, Blobel CP (1997). Evidence for distinct serine protease activities with a potential role in processing the sperm protein fertilin. Dev Biol.

[B017] Marcello MR, Evans JP (2010). Multivariate analysis of male reproductive function in Inpp5b−/− mice reveals heterogeneity in defects in fertility, sperm–egg membrane interaction and proteolytic cleavage of sperm ADAMs. Mol Hum Reprod.

[B018] McLaughlin EA, Frayne J, Barker HL, Jury JA, Jones R, Ford WC, Hall L (1997). Cloning and sequence analysis of rat fertilin alpha and beta--developmental expression, processing and immunolocalization. Mol Hum Reprod.

[B019] NCBI (2020). BLAST®: Basic Local Alignment Search Tool.

[B020] NCBI (2020). Primer-BLAST.

[B021] Oh J, Woo J-M, Choi E, Kim T, Cho B-N, Park ZY, Kim YC, Kim DH, Cho C (2005). Molecular, biochemical, and cellular characterization of epididymal ADAMs, ADAM7 and ADAM28. Biochem Biophys Res Commun.

[B022] Perry ACF, Gichuhi PM, Jones R, Hall L (1995). Cloning and analysis of monkey fertilin reveals novel α subunit isoforms. Biochem J.

[B023] Primakoff P, Hyatt H, Tredick-Kline J (1987). Identification and purification of a sperm surface protein with a potential role in sperm-egg membrane fusion. J Cell Biol.

[B024] Tingari MD, El-Manna MM, Rahim ATA, Ahmed AK, Hamad MH (1986). Studies on camel semen. I. Electroejaculation and some aspects of semen characteristics. Anim Reprod Sci.

[B025] Waters SI, White JM (1997). Biochemical and molecular characterization of bovine fertilin α and β (ADAM 1 and ADAM 2): a candidate sperm-egg binding/fusion complex. Biol Reprod.

[B026] Yuan R, Primakoff P, Myles DG (1997). A role for the disintegrin domain of cyritestin, a sperm surface protein belonging to the ADAM family, in mouse sperm–egg plasma membrane adhesion and fusion. J Cell Biol.

[B027] Zalata A, Atwa A, Mokhtar N, Khaled M (2016). Relationship between fertilin β mRNA expression and the fertilizing potentials of human spermatozoa. Egypt J Biochem Mol Biol.

